# Plasticity of Blood- and Lymphatic Endothelial Cells and Marker Identification

**DOI:** 10.1371/journal.pone.0074293

**Published:** 2013-09-10

**Authors:** Johannes Keuschnigg, Sirkku Karinen, Kaisa Auvinen, Heikki Irjala, John-Patrick Mpindi, Olli Kallioniemi, Sampsa Hautaniemi, Sirpa Jalkanen, Marko Salmi

**Affiliations:** 1 MediCity Research Laboratory, University of Turku, Turku, Finland; 2 Department of Medical Microbiology and Immunology, University of Turku, Turku, Finland; 3 Turku Doctoral Program of Biomedical Sciences, Turku, Finland; 4 Research Programs Unit, Genome-Scale Biology, and Institute of Biomedicine, Biochemistry and Developmental Biology, University of Helsinki, Helsinki, Finland; 5 National Institute of Public Health and Welfare, Turku, Finland; 6 Department of Otorhinolaryngology - Head and Neck Surgery, Turku University Hospital, Turku, Finland; 7 FIMM, Institute for Molecular Medicine Finland, University of Helsinki, Helsinki, Finland; 8 Department of Medical Biochemistry and Genetics, University of Turku, Turku, Finland; University of Miami School of Medicine, United States of America

## Abstract

The distinction between lymphatic and blood vessels is biologically fundamental. Here we wanted to rigorously analyze the universal applicability of vascular markers and characteristics of the two widely used vascular model systems human microvascular endothelial cell line-1 (HMEC-1) and telomerase-immortalized microvascular endothelial cell line (TIME). Therefore we studied the protein expression and functional properties of the endothelial cell lines HMEC-1 and TIME by flow cytometry and *in vitro* flow assays. We then performed microarray analyses of the gene expression in these two cell lines and compared them to primary endothelial cells. Using bioinformatics we then defined 39 new, more universal, endothelial-type specific markers from 47 primary endothelial microarray datasets and validated them using immunohistochemistry with normal and pathological tissues. We surprisingly found that both HMEC-1 and TIME are hybrid blood- and lymphatic cells. In addition, we discovered great discrepancies in the previous identifications of blood- and lymphatic endothelium-specific genes. Hence we identified and validated new, universally applicable vascular markers. Summarizing, the hybrid blood-lymphatic endothelial phenotype of HMEC-1 and TIME is indicative of plasticity in the gene expression of immortalized endothelial cell lines. Moreover, we identified new, stable, vessel-type specific markers for blood- and lymphatic endothelium, useful for basic research and clinical diagnostics.

## Introduction

Abnormal function of blood and lymphatic vessels is important in multiple pathological conditions including inflammation and cancer [1]. Many of these diseases involve dysregulated formation of new vessels, and the endothelial cells play a key role in this neo(lymph)angiogenic process. Lymphatic endothelial cells (LECs) originally derive from embryonic blood endothelial cells (BECs) during embryogenesis. Therefore, it is not surprising that the two endothelial cell types share features such as flat morphology, apico-basal polarity and certain common endothelial-specific proteins. However, many phenotypic and genetic characteristics are unique for one or the other vessel type and are routinely used to differentiate between blood and lymph vessels in pathological specimens and vascular biology. For instance, blood vessels express plasmalemma vesicle associated protein 1 (PV-1), endoglin, neuropilin-1 (NRP-1) and collagen IV [2]. Lymph vessels on the other hand express markers such as podoplanin (PDPN), lymphatic endothelial hyaluronan receptor-1 (LYVE-1), VEGF receptor-3 (VEGFR-3) and prospero related homeobox 1 (PROX-1).

Many of these markers have been identified from microarray studies in which differentially expressed BEC and LEC genes have been analyzed from individual specimens. For instance, microarray analysis of gene expression in cultured lymphatic and blood dermal microvascular endothelial cells showed that <400 genes are differentially expressed [3,4]. Among these PROX-1 is known to be responsible for the induction of several other LEC-specific genes and for the downregulation of numerous BEC-specific genes [4] making it the key regulator of lymphatic cell differentiation [5].

Pathobiological processes involving endothelial cells, such as inflammation, coagulation and neoangiogenesis, are often studied *in vitro*. This requires the isolation and culture of BECs and LECs. However, primary endothelial cells are difficult to isolate (especially from humans) and cannot be cultured for extended periods of time. Therefore, immortalized endothelial cell lines are widely used as a substitute. These cells are usually well characterized, express the accepted markers and can be grown up to passage 40 and beyond. Two cell lines extensively used as models for blood endothelial cells (more than 800 citations in PubMed) are the polyoma-virus transformed human dermal microvascular endothelial cell line-1 (HMEC‑1) [6] and the telomerase-immortalized human microvascular endothelial cell line (TIME) [7]. They have been reported by multiple morphological, phenotypical and functional criteria to phenotypically and functionally represent BECs.

However, we unexpectedly noticed LEC-specific markers in HMEC-1 and TIME. Therefore, we started to systematically examine their hybrid phenotype at protein and gene expression level. In addition, when comparing their gene expressions to those of primary endothelial cells, the heterogeneity of BEC- and LEC-specific gene expression profiles in different published studies became apparent. Hence we generated a novel, more universal database consisting of 47 primary blood- and lymphatic endothelial gene expression profiles. We used this tool to identify universally applicable new BEC and LEC markers. We then showed the validity of this *in silico* approach by testing two exemplary new markers in normal, chronically inflamed and malignant tissues.

## Materials and Methods

### Cell isolation and culture

The original HMCE-1 cell line [6] was donated by Edwin W. Ades from the Center for Disease Control and Prevention (Atlanta, USA) and TIME cells [7] were purchased from ATCC (#CRL-4025). Human umbilical vein endothelial cells (HUVECs) were isolated and cultured as described [8]. Endothelial cells were cultured on plastic without coating. Peripheral blood mononuclear cells (PBMC) and polymorphonuclear cells (PMN) were isolated from healthy donors using Ficoll-Paque Plus and Percoll gradient centrifugation (Amersham Biosciences, Uppsala, Sweden).

### Antibodies

The following primary antibodies were used: PAL-E [9] (mIgG2a, Abcam, Cambridge, UK), anti-CD31 (mAb 2C8 [10], mIgG1), anti-podoplanin (mIgG1, Acris Antibodies, Herford, Germany), anti-LYVE-1 (rabbit IgG, Reliatech, Wolfenbüttel, Germany). Anti-PV-1 antibody (174/2 [11], mIgG1), directly labeled anti-PV‑1 (174/2-FITC [11,12]) and anti-Clever-1 (372 and 372-Alexa 488 [13], common lymphatic endothelial and vascular endothelial receptor-1) were generated in our laboratory. Goat polyclonal Abs against COLEC12 and MCAM were from R&D Systems (R&D Systems, MN, USA).

As negative control antibodies we used AK-1 (mIgG1, In Vivo Biotech Services GmbH, Hennigsdorf, Germany), mIgG2a neg. contr. (R&D Systems), rabbit serum (C12SB, AbD Serotec, Kindlington, UK) and goat serum (Vector Laboratories Inc., Burlingham, CA, USA).

Secondary antibodies were fluorophore-labeled (FITC, PE, Alexa 488 and Alexa 546) anti-mouse IgG (total or isotype specific), anti-rabbit IgG and anti-goat IgG (Sigma Aldrich, Saint Louis, MO, USA and Molecular Probes, Eugene, OR, USA).

### Flow cytometry

Cells were fixed with 4% ice-cold paraformaldehyde and permeabilized using a 0.02% saponin solution. The cells were sequentially incubated with primary and secondary antibodies. Appropriate isotype specific negative controls were used in all stainings. Analyses were performed on a FACSCalibur System using CellQuest Pro software (Becton Dickinson, Franclin Lake, NJ, USA). Median fluorescence intensities of specific stainings (sMFI) were calculated as [sMFI] = [MFI of the antigen specific staining] - [MFI of corresponding negative control].

### Immunohistochemistry

All experiments involving human tissues were approved by the Ethical Committee of the Hospital District of Southwest Finland and written informed consent was obtained. The use of tissues abided by the declaration of Helsinki. Frozen sections of normal human peripheral lymph nodes, inflamed tonsils and bladder cancer as well as colorectal cancer were incubated with anti-COLEC12 and anti-MCAM antibodies followed by incubation with Alexa Fluor 546-coupled second-stage antibodies. In a third step we used directly labeled anti-PV-1 and anti-Clever-1 mAbs, which are established markers for blood [11,14] and lymphatic vessels [13] as well as anti-LYVE-1 and anti-podoplanin antibodies. Stainings were mounted in ProLong Gold containing DAPI (Molecular Probes) and analyzed on a LSM 510 confocal microscope using a Plan Neofluar 20x/0,5 air objective and LSM ZEN software (Carl Zeiss Microimaging, Göttingen, Germany).

### Immunofluorescence

Cells were cultured on glass coverslips (Menzel, Braunschweig, Germany) in 24-well plates. Cells were fixed in 0.2% paraformaldehyde and permeabilized with 0.2% Triton X-100 in PBS for 5 min on ice. After sequential incubation with primary and secondary antibodies cells were mounted as above and analyzed on a LSM 510 confocal microscope using a Plan Neofluar 40x/1.3 Oil-immersion objective and LSM ZEN software.

### In vitro flow assay

The protocol was modified from [15]. HMEC-1, TIME and HUVECs were seeded at 1.25 x 10^5^ cells per channel into ibidi μ-slide VI 0,4 chambers (ibidi GmBH, Martinsried, Germany) coated with ibiTreat and grown to confluence overnight. In the morning, medium was replaced with fresh medium containing 100 U TNF-α ml-1 (for rolling and adhesion experiments) or 500 U TNF-α ml-1 (for transmigration experiments).

At the beginning of the experiment the channel was stabilized for one minute with binding buffer (Dulbecco’s Phosphate Buffered Saline containing 0.1% human serum albumin) using a laminar shear of 0.75 dyn cm^-2^. PBMCs or PMNs (1 x 10^6^ cells ml-1 in binding buffer) were then perfused through the capillary at 0.75 dyn cm-2. Rolling and adherent cells were analyzed two minutes after addition of PBMCs or PMNs to the capillary by recording 5 fields (0.5676 mm^2^ per field) for 15 s each.

For interaction and transmigration studies, PBMCs or PMNs (1 x 10^6^ cells ml-1 in binding buffer) were perfused through the capillary for 5 min at 0.75 dyn cm^-2^ followed by binding buffer (without cells) for 10 min for PMN and 20 min for PBMC at the same laminar shear to allow adherent cells to transmigrate. Interaction and transmigration of PMN were analyzed by recording 5 fields 10 min after perfusion of cells, and for PBMCs the same analyses were done at 20 min after perfusion. Assays were performed with an Olympus IX70 inverted microscope (Olympus, Tokyo, Japan) equipped with a cooled ORCA-R2 CCD camera (Hamamatsu, Hamamatsu City, Japan) using 100x magnification. For each experiment (n=3) different HUVECs as positive control and PBMCs/PMNs isolated from different healthy donors were used.

Analyses were carried out off-line by manual counting. 5 fields per capillary were analyzed for rolling, adherent and transmigrated cells. Cells were defined as rolling if they moved slowly into the direction of flow and as adherent if they remained stationary. At later time points, phase bright cells were counted as adherent and phase dark cells as transmigrated underneath the endothelium. The percentage of transmigrating cells was calculated as [migrating cells]/([adherent cells] + [migrating cells]). The absolute numbers of counted PMN with HUVECs (positive control) per experiment were: 19 ± 3 (mean ± s.e.m.) rolling and 827 ± 24 adherent cells (2 min time point); 1471 ± 206 adherent and 149 ± 34 transmigrated cells (10 min time point). In case of PBMC, 38 ± 3 cells rolled and 323 ± 43 adhered after 2 minutes, and 480 ± 55 cells adhered and 54 ± 13 cells transmigrated at the 20 min time point.

### HMEC-1 and TIME microarray

We tested the effect of the confluency of the cell cultures on the gene expression and found some differences (data not shown). Therefore we standardized our cell cultures and used 80% confluent cultures for our experiments. Total RNA was isolated from ~80% confluent HMEC-1 and TIME cultures using a NucleoSpin RNA isolation kit (Macherey & Nagel, Düren, Germany). Samples were subsequently processed with Affymetrix GeneChip 3’ IVT Express Kit and hybridized to GeneChip Human Genome U133 plus 2.0 Array (all Affymetrix, Santa Clara, CA, USA) at +45°C. All assay steps were performed independently with four biological replicates of each cell type. Samples are deposited in Geo Datasets with the accession number GSE42216.

HMEC-1 and TIME microarrays can be accessed by using the following link:

http://www.ncbi.nlm.nih.gov/geo/query/acc.cgi?token=trwdtegooeoccly&acc=GSE42216

### BEC and LEC gene expression datasets

To analyze differential gene expression between BECs and LECs, 47 publicly available gene expression profiles from the Gene Expression Omnibus (GEO) database [16] were used. The datasets were derived from three different Affymetrix gene expression platforms: Human Genome U133 Plus 2.0 Array (GPL570), Human Genome U133A 2.0 Array (GPL571) and Human Exon 1.0 ST Array (GPL5188). Distribution of samples and cell types over the platforms and grouping into Datasets A and B is shown in Table S1 in File S1. A complete list of the samples including their annotations is available as Table S2 in File S1.

The raw gene expression data for each sample was downloaded from the GEO database [16]. The probes were annotated for their gene identifications from the Ensembl database [17] using a custom probe mapping [18]. The samples from each of the three platforms (GPL570, GPL571, GPL5188) were normalized in subgroups using GC-RMA [19] and their expression values log_2_-transformed. Subsequently, the three datasets representing different gene array versions were combined into a complete expression matrix using the reannotated gene identifications. Genes that did not have expression values in at least 40% of the samples were removed, after which our datasets contained 19081 genes. To account for inter-platform differences we subtracted each gene’s median expression from the gene’s expression values and divided the result with the gene’s standard deviation. This was done for the complete expression matrix that contained all samples from all platforms. The complete expression matrix can be downloaded from http://users.utu.fi/masalmi/Keuschnigg_Suppl_Dataset_I.xlsx.

A multidimensional scaling (MDS) plot (Euclidean distance) was created for Dataset A, HMEC-1, TIME and prostate control samples, which were normalized using GC‑RMA [19]. For ingenuity IPA-software analysis genes that met the cut-off of ≥2-fold change in expression with a multiple hypotheses corrected p-value of ≤0,05 and that were associated with biological functions in the Ingenuity Knowledge Base were considered for the analysis.

### Statistical analyses

Results of the *in vitro* flow experiments are expressed as means ± standard error of mean. Two-tailed student’s t-test with unequal variance was used to evaluate statistical significance. For the microarray analysis p-values were multiple hypotheses corrected using robust false discovery rate estimation [20]. Genes that were differentially expressed with multiple hypotheses corrected p-value ≤ 0.05 and fold change ≥ 2 were considered as significant. For ingenuity IPA analyses, right-tailed Fisher’s exact test was used to calculate the p-values.

## Results

### HMEC-1 and TIME express both lymphatic and blood vascular markers

**Figure 1:**
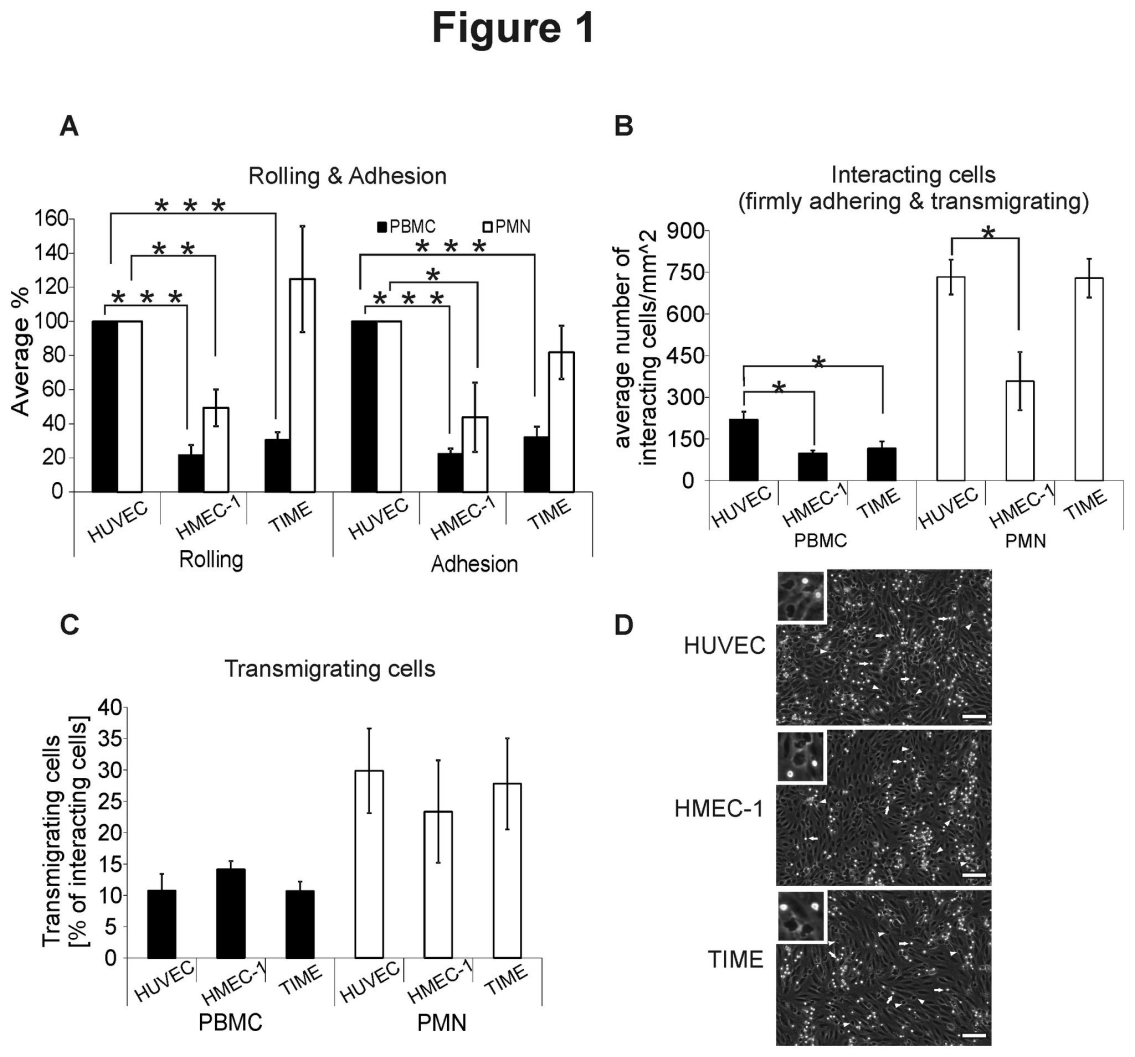
HMEC-1 and TIME express both blood vascular and lymphatic markers. HMEC-1 and TIME cells were stained for flow cytometry using the indicated antibodies against (A) blood vascular markers and (B) lymphatic markers. The marker region shows the antigen-specific staining and is set based on the species- and isotype-specific negative controls. One representative negative control is depicted here. The numbers in the upper right corners of the histograms are the specific median fluorescence intensities of the given antigen, calculated as detailed in materials and methods. (C) Representative images of TIME cells simultaneously expressing lymphatic and blood vascular markers in the same cell are depicted and indicated with white arrows. The scale bar represents 20 µm.

We searched for suitable immortalized endothelial cell lines to be used in inflammation models for human blood vascular endothelium. Based on a plethora of literature we focused on HMEC-1 and TIME cells. Flow cytometric analyses indeed confirmed the expression of established BEC markers PAL-E and CD31 on both cell types (Figure 1A). Both, the anti-PV-1 antibody and PAL-E recognize PV-1, albeit different epitopes [11,12]. The shift of the entire peak when compared to the negative control indicates low-level expression of PV-1 in all cells. Strikingly, however, both HMEC-1 and TIME were also uniformly positive for the lymphatic marker PDPN. In addition, both cell lines also expressed CLEVER-1 and low levels of LYVE-1 (Figure 1B). To confirm the expression of both types of markers in the same cell, we stained TIME cells for microscopy (Figure 1C). Indeed we could show that PV-1 and CD31 are expressed in the same cell as PDPN. Thus, HMEC-1 and TIME cells express a mixture of both vascular and lymphatic cell markers and are therefore clearly distinct from blood endothelial cells.

### Altered leukocyte-endothelial adhesion cascade in HMEC-1 and TIME cells

**Figure 2:**
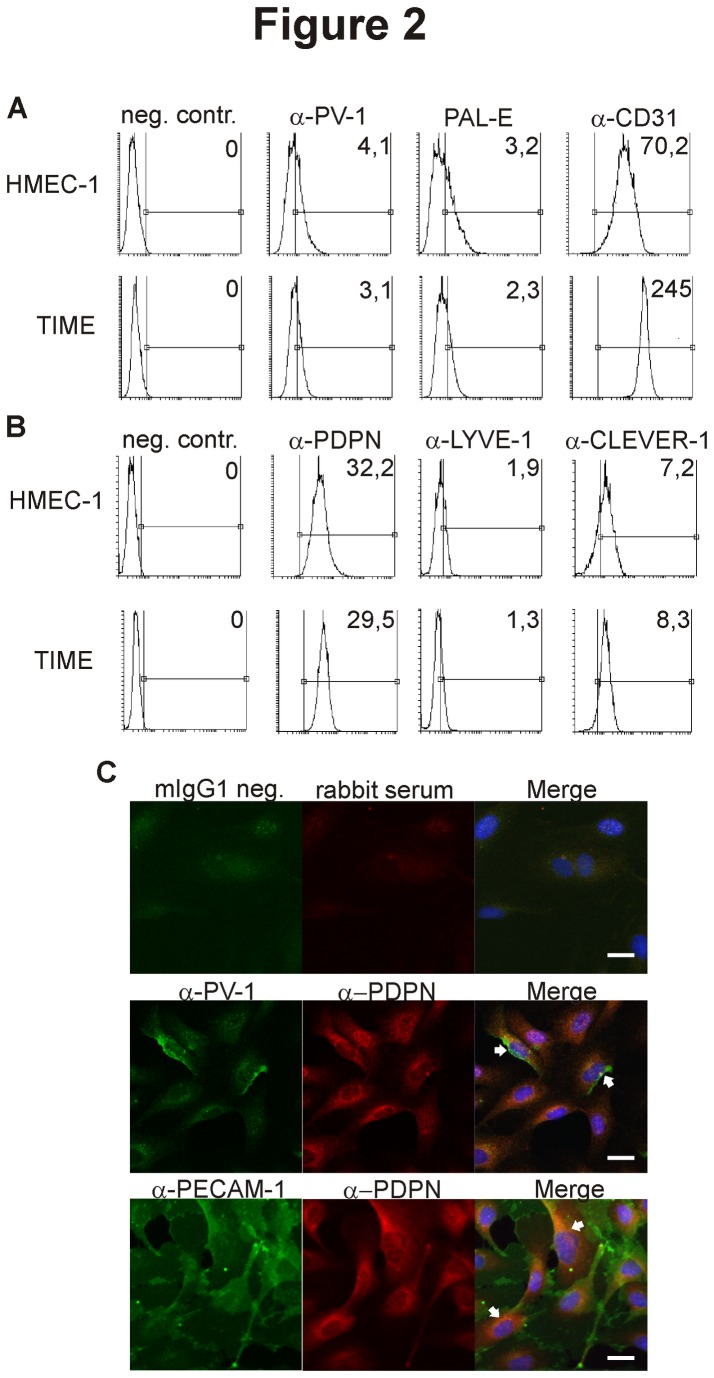
HMEC-1 and TIME show aberrant leukocyte-endothelial interactions under physiological shear stress. (A) Rolling and adhesion of PBMC and PMN on HMEC-1, TIME and HUVEC were analyzed using in vitro flow assay. The results are normalized to HUVEC (100%). (B) Absolute numbers of interacting (firmly adhering and transmigrating) leukocytes and (C) the transmigration percentage (the numbers of transmigrated cells divided by the numbers of interacting cells) on the three endothelial monolayers were determined. Data are shown as mean ± SEM (n = 3 for each assay, each with different leukocyte donors). *P ≤ 0.05. **P ≤ 0.01. ***P ≤ 0.001. (D) Images of representative endothelial monolayers 10 min. after start of PMN transmigration studies are shown. Phase contrast bright cells (representative cells indicated by white arrows) are located on the apical surface of the endothelial cells and phase contrast dark cells (representative cells indicated by white arrow-heads) are situated below the monolayer. Note that all three endothelial types form confluent intact monolayers. Inserts show adhering and transmigrating cells in more detail. Scale bars represent 100 µm.

To study how HMEC-1 and TIME behave in functional assays we analyzed leukocyte-endothelial cell interactions in an *in vitro* flow assay. PBMC rolling and adhesion on both HMEC-1 and TIME were severely impaired when compared to freshly isolated HUVECs (Figure 2A). Similarly, the absolute numbers of interacting PBMC (firmly adhering and transmigrating) were significantly decreased by ~60% on both HMEC-1 and TIME (Figure 2B).

Extravasation of PMN across TIME monolayers occurred with similar efficiency as on HUVECs. On the other hand, both rolling and adhesion of PMN on HMEC‑1 were significantly impaired (Figure 2A). Furthermore, the absolute numbers of PMN interacting with HMEC-1 were significantly decreased by >50% (Figure 2B).

However, once firmly adhered, comparable percentages of PBMC and PMN transmigrated across HUVEC, HMEC-1 and TIME monolayers. Thus, HMEC-1 and TIME cells differ from pure endothelial HUVECs in their capacity to support several steps of leukocyte extravasation.

### Genome wide expression profiling of HMEC-1 and TIME places them near to BECs and LECs

**Figure 3:**
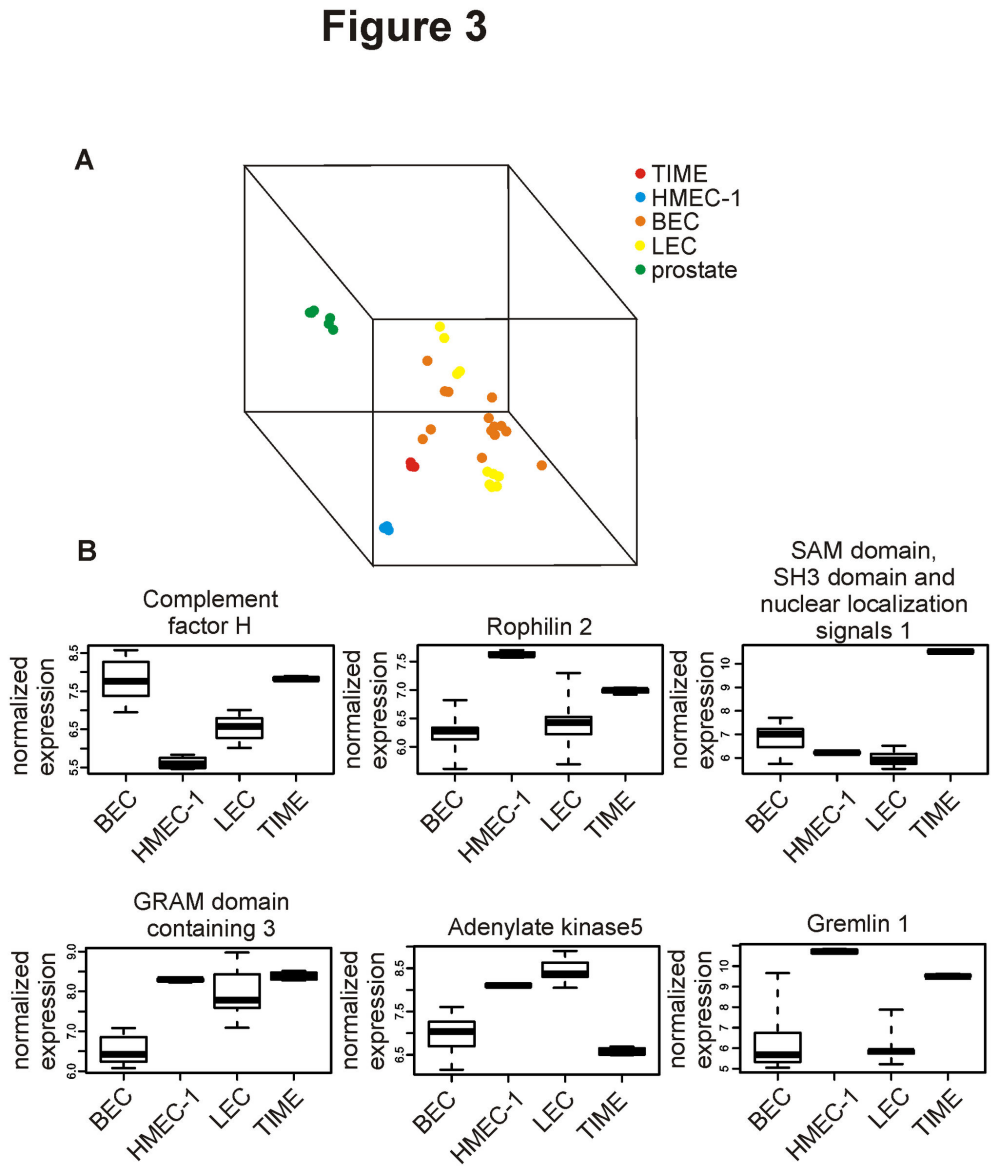
Gene expression profiles of HMEC-1 and TIME reveal differences from pure BEC and LEC. (A) A multidimensional scaling plot (MDS-plot) showing clustering of TIME, HMEC-1, primary BEC and LEC (and prostate tissue as a control) based on the expression of about 19000 genes. (B) Exemplary genes that are differently expressed in HMEC-1 and/or TIME when compared to BEC and LEC in the normalized Dataset A. Data is presented as mean with the middle quartile and minimum/maximum values.

To get a more global view on the phenotypic alterations in HMEC-1 and TIME, gene expression analyses were performed. We subjected resting HMEC-1 and TIME cells to Affymetrix microarray analyses and compared the results to BEC- and LEC microarray data available in the public domain (Geo DataSets [16]). For that purpose we combined 14 BEC and 10 LEC datasets from the same platform as our own HMEC-1 and TIME analysis (GPL570) to produce Dataset A. MDS is a visualization methodology that aims at finding similarities between data points [21]. Here the 3D-MDS plot clusters biological samples according to their genome-wide expression profiles. Prostate tissue was included for an unbiased comparison of distances between HMEC-1, TIME, BEC and LEC samples. As illustrated in Figure 3A and the Movie S1, TIME and HMEC‑1 cluster near to, but not among primary endothelial cells. They appear to be somewhat more closely related to BECs than to LECs. Moreover, the expression of several genes was up- or down-regulated in HMEC-1 and TIME in comparison to primary BEC and LEC (Figure 3B) in the normalized Dataset A. There were also notable differences in the expression of some genes (such as complement factor H, adenylate kinase 5 and SAM domain, SH3 domain and nuclear localizations signals 1), between HMEC-1 and TIME. Thus, also the gene expression patterns in HMEC-1 and TIME are clearly different from those of pure BECs.

### Search for new BEC/LEC markers using publicly available microarray data

As microarray analyses often rely on cells freshly isolated from one or a few individuals, one drawback of these analyses is usually the limited biological variability. We therefore hypothesized that analyses of numerous pooled BEC- and LEC microarray datasets could result in the discovery of previously unknown, differentially expressed genes and thus more universally applicable BEC/LEC markers. Even though combining datasets from different parts of the vascular tree [22,23] results in the loss of vascular bed-specific information, it is the only way to increase the possibility to find true BEC/LEC specific markers when analyzing dozens of microarrays created by several laboratories.

We used our Dataset A (14 BEC and 10 LEC from the same platform) for the initial comparisons. In addition, we queried the NCBI GEO DataSets [16] database and assembled a larger dataset of 33 BEC and 14 LEC (Dataset B). These 47 microarray experiments with primary cells come from 12 separate experiments run on three different Affymetrix platforms. In addition we compared our results with two previously published studies, which have specifically aimed at reporting differently expressed genes in BEC and LEC [3,4].

**Figure 4:**
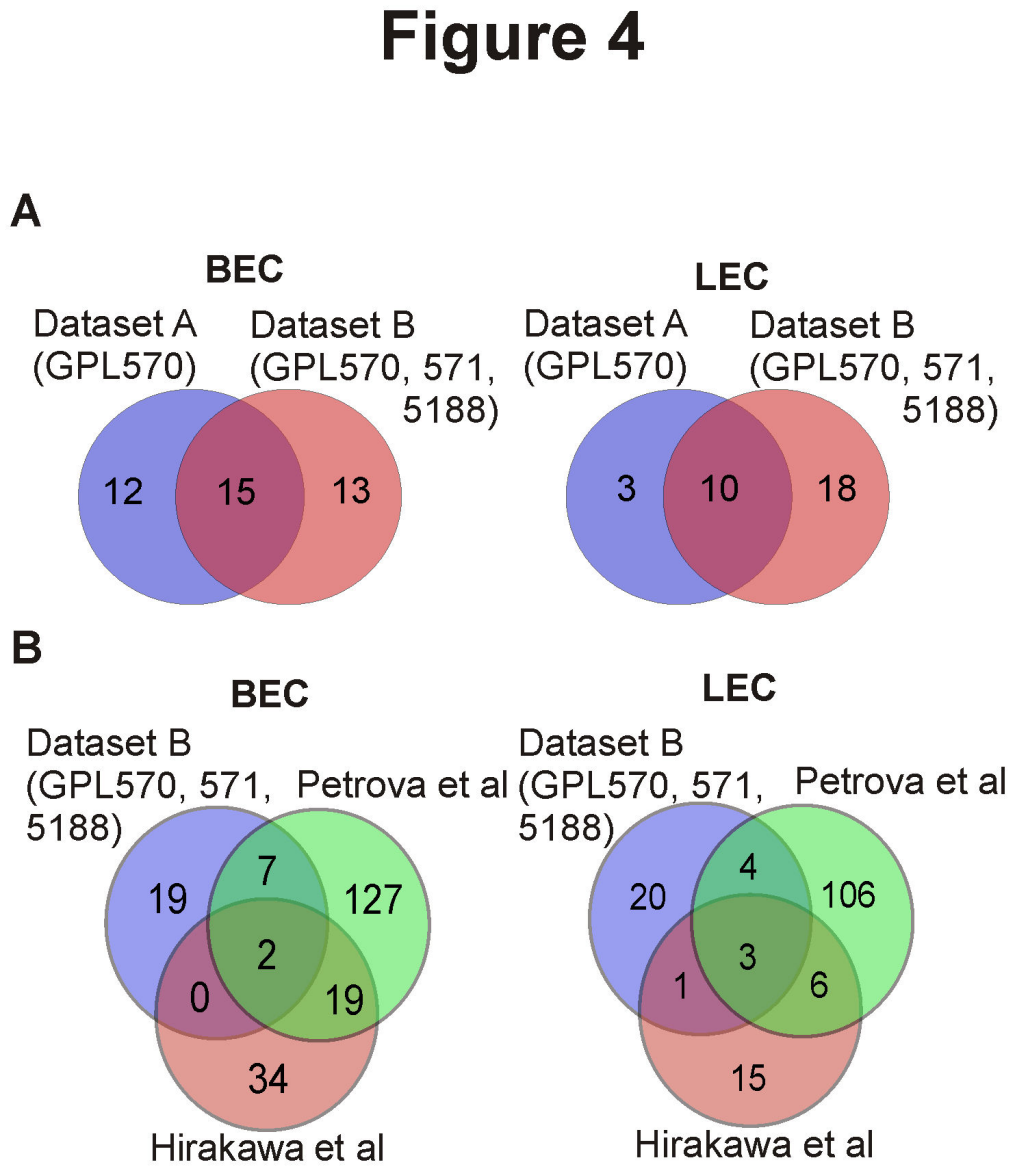
Identification of BEC and LEC specific genes in different individual- and pooled microarray analyses. (A) The numbers of BEC and LEC specific genes obtained from analyses of a restricted Dataset A (24 samples, one platform: GPL570) and of an extensive Dataset B (47 samples, three platforms: GPL570, GPL571 and GPL5188) are shown using Venn-diagrams. (B) Similar comparisons were performed between our extensive Dataset B and two individual studies (Hirakawa and Petrova) reporting BEC- and LEC specific genes.

Comparison of the smaller Dataset A with the extensive Dataset B exhibits only a limited overlap, when using a threshold of ≥2-fold change in expression levels with a multiple hypotheses corrected p-value of ≤0,05 to define a differentially expressed gene. For BEC-specific molecules, Dataset A revealed 27 and Dataset B 28 genes with 15 genes being common to both analyses. For LEC-specific molecules, 13 were identified from Dataset A, 28 from Dataset B, with 10 genes being LEC-specific in both datasets (Figure 4A).

Comparison of our analysis of Dataset B to previous analyses performed by Hirakawa et al [3] and Petrova et al [4] also revealed very little overlap (Figure 4B). In BECs only two and in LECs three genes were common to all three studies. All three analyses found the prototype LEC markers podoplanin and PROX-1 to be specific for LECs. The third LEC-specific protein was reelin [24] (RELN). In contrast, none of the commonly used BEC markers was scored as BEC-specific in all three studies. Surprisingly, both genes consistently found to be BEC-specific were neuronal cell adhesion molecule [25] (NRCAM) and chemokine ligand 1 [26] (CXCL1; Table 1, Table S3 in File S1).

**Table 1:** BEC- or LEC-specific genes in different microarray studies.

**Blood endothelial cell specific genes**							
Intersection:	Keuschnigg	Keuschnigg	Keuschnigg	Petrova	Keuschnigg	Petrova	Hirakawa
	Petrova	Petrova	Hirakawa	Hirakawa			
	Hirakawa						
Number of genes:	2	7	0	19	19	127	34
Genes:	NRCAM	TAGLN		CD44	FSTL1	IL8	AUTS2
	CXCL1	RNASE1		LTBP2	MCAM	AXL	DSG1
		CAP2		ITGA5	ZNF207	FAP	ITGA4
		PLA2G4A		BMP6	VAMP8	ACTA2	GPR39
		ISG15		VCAN	RGS5	KRT7	COL6A3
		DKK1		CXCR4	SEC 61B	LPHN2	IL13RA2
		IFI27		SRGN	GLIPR1	SELP	FBLN5
				IL4R	SH3BP4	TPM2	BMP1
				FLT1	EMP3	SERPINE1	CSF2RB
				PCDH1	TSPAN3	IGF2BP3	ITGB3
				VEGFC	HOXB2	PLAU	VWF
				CDH2	JAM3	PFN2	F2R
				COL1A2	NCL	CHST1	ESM1
				PGF	RP3-523C21.1	MMP1	EFEMP2
				CCL2	CCT5	BASP1	CD93
				C17orf72,ICAM2	SHISA3	MLLT11	SPARC
				ITGB5	MCTP1	CLU	LAMC1, LAMB2
				CCRL2	GATA6	TGFBI	PECAM1
				COL6A1	C1orf54	IL6	FN 1
						TRIM22	LAMB1
						*	*
**Lymphatic endothelial cell specific genes**							
Intersection:	Keuschnigg	Keuschnigg	Keuschnigg	Petrova	Keuschnigg	Petrova	Hirakawa
	Petrova	Petrova	Hirakawa	Hirakawa			
	Hirakawa						
Number of genes:	3	4	1	6	20	106	15
Genes:	PROX1	GMFG	CEACAM1	CXCL12	PVRL3	RBP1	F2RL1
	PDPN	FABP4		MRC1,MRC1L1	TNFSF10	MAF	LGALS8
	RELN	PPARG		CALCRL	HSD17B2	CH25H	PSMG1
		MGP		TFF3	CD36	SEPP1	CCL5
				ANGPT2	ID1	SLC26A4	JUP
				DSP,SNRNP48	ADRB1	RGS16	GLRB
					NID1	ITGA9	THBS1
					LAYN	CDKN1C	HMMR
					ADAMTSL3	CRMP1	IL6ST
					SNAI2	PCSK6	JAG1
					EPS8	ITGA1	CCL20
					AK5	MEF2C	TGFA
					RP11-65D13.1	APOD	FGF12
					FDFT1	PDLIM3	ITGA6
					GRAMD3	CCNE2	MFAP3
					COLEC12	TIMP3	
					GHR	CD200	
					HEY1	ADD3	
					GYPC	TK1	
					TFPI	LIPA	
						*	

Results from the individual studies are marked by the name of the first authors. The terms “intersection” and “number of genes” refer to the overlaps between the individual studies in the venn diagrams in Figure 4B.

* Indicates truncation of gene list after the top 20 genes. For a complete list see Table S3 in File S1.

Further analysis of the results using IPA software (Ingenuity^®^ Systems, www.ingenuity.com) showed that both LEC and BEC specific genes were mostly involved in processes such as cardiovascular system development, tissue development and organ development (p-values ranging from ≤0,05 to ≤0,0001), supporting their potential relevance to cell differentiation.

### Two new markers MCAM and COLEC12 are useful for the distinction between blood and lymphatic endothelium in normal, chronically inflamed and cancerous tissues

**Figure 5:**
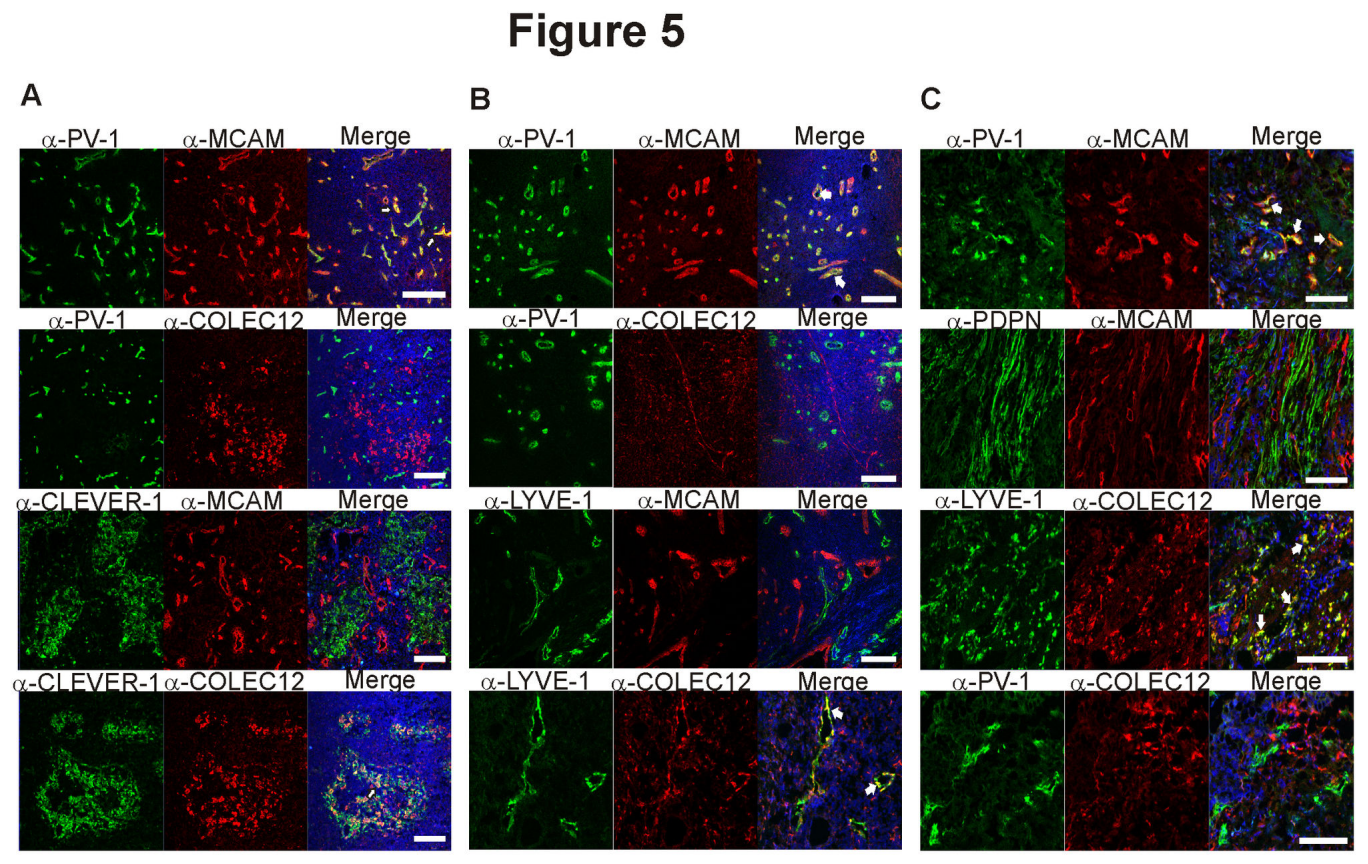
MCAM and COLEC12 are novel BEC- and LEC- specific markers. Two new endothelial markers MCAM and COLEC12 (selected from our analysis of Dataset B) were used to stain (A) normal human lymph nodes together with two established vascular markers PV-1 (for BEC) and CLEVER-1 (for LEC). In addition, (B) chronically inflamed tonsils and (C) specimens from bladder cancer and colorectal cancer were stained with the antibodies against the indicated proteins. LYVE-1 and COLEC12 co-staining was done on colorectal cancer specimens, whereas the other stainings represent bladder cancer. MCAM staining colocalized very well with the established BEC marker PV-1 whereas no colocalization could be detected between MCAM and the established LEC markers LYVE-1 and podoplanin. COLEC12-staining on the other hand showed colocalization with LYVE-1 but not PV-1. White arrows point to areas of colocalization. Nuclear counterstaining was performed with DAPI. Scale bars represent 100 µm.

Using the list of new blood- and lymphatic endothelium specific molecules derived from analyses of Dataset B we chose the proteins melanoma cell adhesion molecule (MCAM) as a new BEC-marker and collectin placenta 12 (COLEC12) as LEC-marker to test whether our *in silico* findings could also be confirmed in an independent manner. To that end we examined how the new markers relate to the established vascular marker PV-1 [12] and lymphatic markers CLEVER-1 [13] (common lymphatic endothelial and vascular endothelial receptor-1), LYVE-1 and podoplanin in immunohistochemical stainings (Figure 5). The new BEC marker MCAM colocalized very well with the established blood vascular marker PV-1 (the epitope of the widely used antibody PAL-E; pathologische anatomie leiden-endothelium [12]), while there was no overlap with the staining pattern of the LEC markers CLEVER-1 and podoplanin. Similarly, the new LEC marker COLEC12 showed the expected staining pattern with colocalization with CLEVER-1 and LYVE-1 and mutually exclusive staining with PV-1. Thus, our new *in silico* derived list of BEC- and LEC specific genes should be useful for identification of new markers with a wide application range.

## Discussion

Based on protein expression, gene expression and functional analyses this study shows that the two widely used endothelial cell lines HMEC-1 and TIME exhibit a hybrid phenotype different from pure blood vascular or lymphatic endothelial cells. Furthermore, combination of a comprehensive set of publicly available microarray datasets from primary endothelial cells allowed us to identify novel BEC and LEC markers. This *in silico* approach was feasible for discovery of novel cell-type specific molecules since two representative candidates were successfully validated in independent biological experimentation using normal, inflamed and malignant tissues.

HMEC-1 and TIME are two notable examples of immortalized blood vascular models that have been widely used (their original publications have been cited over 800 times). Both were generated from dermal microvascular endothelial cells without apparent selection for blood vascular endothelial cells [6,7]. As the human microvasculature consists of both BECs and LECs, immortalization without enrichment for one cell type could create a mixed cell line with two different cell populations. Nevertheless, our analyses unambiguously showed that both HMEC-1 and TIME only contain one homogenous population simultaneously expressing markers for both BECs and LECs. During vascular development, both BEC- and LEC markers are transiently present in the same cell and recently it was discovered that LECS also express the blood endothelial fate regulators chicken ovalbumin upstream transcription factor and NOTCH [27]. These findings suggest a delicate balance where minor variations in the expression of the cell fate regulators cause major changes in the endothelial cell differentiation.

Immortalization of primary endothelial cells causes a significant change in their gene expression profile [28]. Furthermore, it can cause changes in the functional properties such as response to cytokines and migration of peripheral blood mononuclear cells across endothelial cells [29]. Interestingly, not only artificially manipulated cells can exhibit modified phenotypes and genotypes, as primary HUVECs for example have been shown to adopt a lymphatic-like phenotype in *in vitro* angiogenesis assays [30]. Previous studies have also demonstrated a loss of lineage specific markers in tissue culture [31] and the importance of extracellular matrix environment for maintaining the cell identity [32]. This well-established plasticity of blood- and lymphatic endothelial cells might explain why HMEC-1 and TIME exhibit a mixed phenotype.

Both HMEC-1 and TIME showed abnormal interactions with leukocytes in functional assays. This is in line with a previous report demonstrating impaired induction of adhesion molecules on HMEC-1 and TIME as well as absence of rolling. However, the endothelial monolayers used in these experiments appeared to be subconfluent [33]. With our confluent monolayers (Figure 2D) we clearly showed that rolling, firm adhesion and transmigration of both lymphocytes and granulocytes do take place with HMEC-1 and TIME, albeit there were quantitative differences in comparison to pure BEC. However, these abnormalities seem to be restricted to early events during the leukocyte extravasation cascade (namely rolling and adhesion). Once leukocytes firmly adhered, the percentages of cells that also transmigrated across the endothelium were comparable between HUVECs, HMEC-1 and TIME. Aberrations in adhesion molecule expression and functions of HMEC-1 and TIME have been first mentioned already in 2004 [34] and 2007 [33]. Surprisingly, these cells have remained widely used models for blood vasculature [35,36]. Our comprehensive characterization of both HMEC-1 and TIME now clearly indicates that they are poorly suited as BEC models.

In our analyses we used HUVECs, a widely used model system [37–40] for the comparison of leukocyte transmigration. These cells are embryonic macrovascular endothelial cells and exhibit only limited similarity with adult microvascular endothelial cells, the cell type across which most of the leukocyte transmigration occurs. As microvascular endothelial cells facilitate the majority of leukocyte transmigration, our results using macrovascular endothelial cells as a reference material might actually underestimate the impaired capacity for leukocyte transmigration exhibited by the microvascular endothelial cell lines HMEC-1 and TIME. However, once activated, HUVECs do allow leukocytes to adhere and transmigrate with similar efficiency as adult dermal microvascular endothelial cells do [33].

As stainings and *in vitro* flow assays concentrate only on a limited number of proteins, we performed microarray analyses to investigate the genome wide expression profiles of HMEC-1 and TIME and compared them to those of primary BECs and LECs. In global expression profiling (MDS-plots), TIME and HMEC-1 cluster distantly from each other, but both endothelial cell lines seem to be relatively closely related to normal BECs. However, this clustering is based on the expression of ≥19000 genes. Considering the fact that only a minor fraction of these genes (<400) seems to be differentially expressed between BEC and LEC [3,4], this result is expected and likely represents an overestimation of the relatedness. Indeed, when analyzing individual genes, it was obvious that both HMEC-1 and TIME express several genes not typically found in endothelial cells.

These findings once more demonstrate the differences between immortalized endothelial cell lines and primary cells. In addition, *in vitro* systems can never fully reproduce the situations predominant in living organisms. Taken together, these points emphasize the need for validation of data using primary endothelial cells and *in vivo* models.

While analyzing the gene expression profiles of primary BEC and LEC in the literature, it became apparent that there is surprisingly little overlap between individual published analyses of BEC- and LEC-specific gene expressions. Hence we utilized our pooled microarray dataset for the identification of novel, universal BEC- and LEC-specific gene expression profiles from primary endothelial cells. Biological sample heterogeneity and differences in cell isolation protocols and preprocessing may account for some variability. For instance, to isolate LECs, Hirakawa et al. [3] used the criteria of CD34^-^, CD31^+^, while Petrova et al. [4] used CD31^-^, PDPN^+^ cells. Furthermore, the threshold for genes to be considered as BEC/LEC specific was set as increase in gene expression with p≤0,002 in the first publication and as 2-fold change in expression levels in the second study. Despite their different analysis criteria, both groups report <400 genes to have changed at least 2-fold between BECs and LECs.

Introduction of biological variability clearly affects the results, since in our analyses the 40% increase in the sample size in Dataset B resulted in the doubling of genes found to be specific for LECs. However, most of the genes present in the restricted Dataset A were also present in the extensive sample set B. This most probably reflects the rather homogenous nature of LECs. In any case, it is noteworthy that none of the commonly accepted BEC markers (e.g. PV-1, endoglin, FVIII) were common to all three analyses. Thus, great caution should be exercised when alluding to the discovery of global cell-type specific markers using low numbers of biological samples.

While the identification of the prototype LEC markers PROX-1 and podoplanin strongly supports our results, the other common genes, RELN, NRCAM and CXCL1 are not usually used for discrimination between BEC and LEC. RELN was found to play a role in radial migration of cortical neurons and maturation of dendrites and was shown to be involved in NOTCH-signaling [41]. The importance of NOTCH-signaling for BEC/LEC specialization could explain the finding of RELN as LEC marker. NRCAM is a neuronal cell adhesion molecule and during neural development it is for example involved in cell proliferation, differentiation and axon growth and guidance (reviewed in [42]). NRCAM has been shown to interact with laminin [43], a protein found to be BEC specific [3]. Interestingly, NRCAM has also been found to associate with neuropilin-2 [44], the co-receptor of the LEC marker VEGFR-3 [2]. CXCL1 on the other hand was recently shown to be induced by prostaglandin E2 and to function in angiogenesis by stimulating microvascular endothelial cell migration and tube-formation [45]. Thus, as is the case with established LEC and BEC markers, none of the new markers is endothelium specific [46–49], but among endothelial subtypes they selectively seem to be expressed either on BEC or LEC.

We confirmed the specificity of representative novel markers with biological samples. MCAM and COLEC12 showed very strong colocalization with the established BEC and LEC markers in immunohistological stainings of human lymph nodes (Figure 5A), chronically inflamed tonsils (Figure 5B) and malignant tissues (Figure 5C). Notably, neither of them was identified by Hirakawa et al. [3] or Petrova et al [4] as endothelium-subtype specific molecules. Nevertheless, it should be noted that MCAM has been previously found to be expressed on haematopoietic cells [50] and tumor cells [51], where it plays a role in the interaction with vascular endothelial cells. In addition, MCAM expression has also been identified on endothelial cells [52,53]. However, its usefulness for the distinction between vessels of blood- and lymphatic origin is new and currently MCAM is not commonly used as endothelial marker.

In conclusion, we demonstrate that the widely used endothelial cell lines HMEC-1 and TIME are not representatives of microvascular blood vascular phenotype, but instead are hybrid cells with both vascular and lymphatic characteristics. Furthermore we show that BEC- and LEC specific gene expression profiles in primary cells differ greatly between individual analyses and that it is possible to identify new universally applicable BEC and LEC-specific molecules through analyses of pooled microarray data.

## Supporting Information

File S1 **Supporting files.** Table S1, Distribution of datasets and cell types across Affymetrix gene expression platforms and inclusion in Dataset A and/or B. Table S2, List of microarray datasets used in this study including source information as provided in NCBI’s GeoDatasets. The original entries in GeoDatasets can be found online by following the hyperlinks underlying each sample Id. Table S3, BEC- or LEC-specific genes in different microarray studies.
(DOC)

Movie S1 **3D MDS plot of BEC, LEC, HMEC-1 and TIME clustering.** A 3-dimensional animation of the clustering of BEC, LEC, HMEC-1 and TIME samples according to their genome wide gene expression. Prostate tissue allows an unbiased comparison of distances between the individual samples.
(WMV)
