# Correction: Plasticity of Blood- and Lymphatic Endothelial Cells and Marker Identification

**DOI:** 10.1371/annotation/cfda269a-cb46-4735-b87f-42afccb6aeb1

**Published:** 2013-10-15

**Authors:** Johannes Keuschnigg, Sirkku Karinen, Kaisa Auvinen, Heikki Irjala, John-Patrick Mpindi, Olli Kallioniemi, Sampsa Hautaniemi, Sirpa Jalkanen, Marko Salmi

Figures 1 and 2 were incorrectly switched. The image currently appearing as Figure 1 belongs with the title and legend for Figure 2, and the image currently appearing as Figure 2 belongs with the title and legend as Figure 1. The titles and legends themselves are in correct order. 

