# Supplementary material for: Plasticity of Blood- and Lymphatic Endothelial Cells and Marker Identification
Source: PLoS One. 2013 Sep 10;8(9):e74293. doi: 10.1371/journal.pone.0074293 (PMC3769239; doi:10.1371/journal.pone.0074293)
Supplement: File S1 — Supporting files. Table S1, Distribution of datasets and cell types across Affymetrix gene expression platforms and inclusion in Dataset A and/or B. Table S2, List of microarray datasets used in this study including source information as provided in NCBI’s GeoDatasets. The original entries in GeoDatasets can be found online by following the hyperlinks underlying each sample Id. Table S3, BEC- or LEC-specific genes in different microarray studies. (DOC) [file pone.0074293.s001.doc]

Table S1. Distribution of datasets and cell types across Affymetrix gene expression platforms and inclusion in Dataset A and/or B.

| Affymetrix platform | Dataset A | Dataset B | Number of samples | Number of BECs | Number of LECs |
| --- | --- | --- | --- | --- | --- |
| Human Genome U133 Plus 2.0 Array (GPL570) | X | X | 24 | 14 | 10 |
| Human Genome U133A 2.0 Array (GPL571) |  | X | 12 | 12 | - |
| Human Exon 1.0 ST Array (GPL5188) |  | X | 11 | 7 | 4 |

**Table S2: List of microarray datasets used in this study including source information as provided in NCBI’s GeoDatasets.** The original entries in GeoDatasets can be found online by following the hyperlinks underlying each sample Id.

| Group | Source | Sample IDs |
| --- | --- | --- |
| BEC | HUVEC | [GSM141250](http://www.ncbi.nlm.nih.gov/geo/query/acc.cgi?acc=GSM141250), [GSM141251](http://www.ncbi.nlm.nih.gov/geo/query/acc.cgi?acc=GSM141251), [GSM272863](http://www.ncbi.nlm.nih.gov/geo/query/acc.cgi?acc=GSM272863), [GSM272864](http://www.ncbi.nlm.nih.gov/geo/query/acc.cgi?acc=GSM272864), [GSM272865](http://www.ncbi.nlm.nih.gov/geo/query/acc.cgi?acc=GSM272865), [GSM344778](http://www.ncbi.nlm.nih.gov/geo/query/acc.cgi?acc=GSM344778), [GSM344779](http://www.ncbi.nlm.nih.gov/geo/query/acc.cgi?acc=GSM344779), [GSM344780](http://www.ncbi.nlm.nih.gov/geo/query/acc.cgi?acc=GSM344780), [GSM472935](http://www.ncbi.nlm.nih.gov/geo/query/acc.cgi?acc=GSM472935), [GSM476782](http://www.ncbi.nlm.nih.gov/geo/query/acc.cgi?acc=GSM476782), [GSM476785](http://www.ncbi.nlm.nih.gov/geo/query/acc.cgi?acc=GSM476785) |
| BEC | Blood vessel endothelial cells | [GSM410678](http://www.ncbi.nlm.nih.gov/geo/query/acc.cgi?acc=GSM410678), [GSM410679](http://www.ncbi.nlm.nih.gov/geo/query/acc.cgi?acc=GSM410679), [GSM410680](http://www.ncbi.nlm.nih.gov/geo/query/acc.cgi?acc=GSM410680), [GSM410681](http://www.ncbi.nlm.nih.gov/geo/query/acc.cgi?acc=GSM410681), [GSM410682](http://www.ncbi.nlm.nih.gov/geo/query/acc.cgi?acc=GSM410682), [GSM410683](http://www.ncbi.nlm.nih.gov/geo/query/acc.cgi?acc=GSM410683), [GSM345269](http://www.ncbi.nlm.nih.gov/geo/query/acc.cgi?acc=GSM345269) |
| BEC | Lung microvascular blood endothelial cells | [GSM580020](http://www.ncbi.nlm.nih.gov/geo/query/acc.cgi?acc=GSM580020) |
| BEC | Dermal microvascular blood endothelial cells | [GSM580013](http://www.ncbi.nlm.nih.gov/geo/query/acc.cgi?acc=GSM580013), [GSM580021](http://www.ncbi.nlm.nih.gov/geo/query/acc.cgi?acc=GSM580021), [GSM580022](http://www.ncbi.nlm.nih.gov/geo/query/acc.cgi?acc=GSM580022), [GSM580023](http://www.ncbi.nlm.nih.gov/geo/query/acc.cgi?acc=GSM580023), [GSM580024](http://www.ncbi.nlm.nih.gov/geo/query/acc.cgi?acc=GSM580024) |
| BEC | Cavernosal endothelial cells | [GSM272854](http://www.ncbi.nlm.nih.gov/geo/query/acc.cgi?acc=GSM272854), [GSM272859](http://www.ncbi.nlm.nih.gov/geo/query/acc.cgi?acc=GSM272859), [GSM272860](http://www.ncbi.nlm.nih.gov/geo/query/acc.cgi?acc=GSM272860), [GSM272861](http://www.ncbi.nlm.nih.gov/geo/query/acc.cgi?acc=GSM272861), [GSM272862](http://www.ncbi.nlm.nih.gov/geo/query/acc.cgi?acc=GSM272862), [GSM272866](http://www.ncbi.nlm.nih.gov/geo/query/acc.cgi?acc=GSM272866), [GSM272867](http://www.ncbi.nlm.nih.gov/geo/query/acc.cgi?acc=GSM272867), [GSM272868](http://www.ncbi.nlm.nih.gov/geo/query/acc.cgi?acc=GSM272868), [GSM272870](http://www.ncbi.nlm.nih.gov/geo/query/acc.cgi?acc=GSM272870) |
| LEC | Dermal microvascular lymphatic endothelial cells | [GSM130277](http://www.ncbi.nlm.nih.gov/geo/query/acc.cgi?acc=GSM130277), [GSM580011](http://www.ncbi.nlm.nih.gov/geo/query/acc.cgi?acc=GSM580011), [GSM580012](http://www.ncbi.nlm.nih.gov/geo/query/acc.cgi?acc=GSM580012) |
| LEC | Lymphatic endothelial cells | [GSM143717](http://www.ncbi.nlm.nih.gov/geo/query/acc.cgi?acc=GSM143717), [GSM143898](http://www.ncbi.nlm.nih.gov/geo/query/acc.cgi?acc=GSM143898), [GSM143900](http://www.ncbi.nlm.nih.gov/geo/query/acc.cgi?acc=GSM143900), [GSM410666](http://www.ncbi.nlm.nih.gov/geo/query/acc.cgi?acc=GSM410666), [GSM410667](http://www.ncbi.nlm.nih.gov/geo/query/acc.cgi?acc=GSM410667), [GSM410668](http://www.ncbi.nlm.nih.gov/geo/query/acc.cgi?acc=GSM410668), [GSM410669](http://www.ncbi.nlm.nih.gov/geo/query/acc.cgi?acc=GSM410669), [GSM410670](http://www.ncbi.nlm.nih.gov/geo/query/acc.cgi?acc=GSM410670), [GSM410671](http://www.ncbi.nlm.nih.gov/geo/query/acc.cgi?acc=GSM410671) |
| LEC | Lung microvascular lymphatic endothelial cells | [GSM580009](http://www.ncbi.nlm.nih.gov/geo/query/acc.cgi?acc=GSM580009), [GSM580010](http://www.ncbi.nlm.nih.gov/geo/query/acc.cgi?acc=GSM580010) |

**Table S3. BEC- or LEC-specific genes in different microarray studies.**

| BEC | Petrova | AGRN, ALDH2, ANPEP, ANXA3, ANXA6, APBB2, ARHGAP22, ARHGDIB, ARL4C, ARL6IP5, ATP1B1, BCAP31,SLC6A8, BGN, BTG2, CAPG, CD59, CDC42EP3, CDKN1A, CLDN7, COL8A1, CTSC, DDX42, DFNA5, ECE1, EIF2B2, ENO2, EPS8, EXT1, FBXL2, FHL2, GADD45A, GBP2, GLCE, GPR126, HMOX1,MCM5,TOM1, ICAM1, IER3, IL32, ITGAV, ITM2A, JUN, KLHL21, LAMA5, LAMC2, LAMP2, LDHB, LOXL2, LPXN, LY75, LYL1, MAPKAPK3, MBOAT7, MMP14, MT1A, MT1B, MT1E, MT1F, MT1H, MT1L, MT3, MVP, NISCH, NMT2, NNMT, NPTX1, NR2F2, NUAK1, PDLIM4, PFKM, PKIA, PLOD1, PLOD2, PLTP, PRKACB, PRNP, PROCR, RAC2, RCN1, RGS10, RGS4, RHOB, RRAS, SELE, SGSH, SLC1A1, SLC3A2, SLC7A7, SMAD3, SMURF2, SORD, SRPX, SRPX2, STAT6, TACSTD2, TANK, TCN2, TFEC, TGM2, TNFRSF21, TP53I3, TPM1, TRAM2, TXNRD2, UAP1, UCHL1, UPP1, ZNF238 |
| --- | --- | --- |
|  | Hirakawa | JAG2, PDGFA, ENG, CD151, COL4A2, COL5A2, PDGFB, CDH13, HSPG2, CDH5, TNFRSF10B, VEGFB, IGF2R |
| LEC | Petrova | PVRL3, AURKB, CDK1, GCH1, LAMP3, USP13, DHCR24, ARRB2, SOCS2, PPAP2A, KIF11, RAMP3, NPTX2, BTBD3, PPP1R2, HLA-DPB1, PEG10, TUBA4A, DUSP5, NOS2, RAPGEF5, DYRK3, CCNB1, DCLK1, NDC80, TOP2A, LDB2, KIAA0101, CEP68, PRKCZ, PDE8A, ALDH1A1, LMNB1, CYP1A1, SPAG5, HIST1H4C, CCNA2, GAB1, TTK, NR2F1, FLT4, CDKN3, LMO2, RAB31, BUB1, SLC39A14, CSRP2, ISG20, GINS1, EPB49, MCM6, ACSL3, PSRC1, TBXA2R, PDLIM1, PYGL, GRAP, MYBL2, KALRN, PTTG1, SMC4, SPRY1, KIF2C, HMGB2, IL7, CETP, PLK1, CENPF, CREM, UBE2C, NDRG1, BUB1B, TBX1, RBPMS, PNP, HOXD10, FOXM1, IGFBP2, MAOA, CTDSPL, CCNB2, LBR, IQGAP2, GPRC5B, RAMP2, EMP2 |

A complete list of genes identified by the studies of Petrova et al and Hirakawa et al excluding the genes present in table 1.
